# Identification of the domains of cauliflower mosaic virus protein P6 responsible for suppression of RNA silencing and salicylic acid signalling

**DOI:** 10.1099/vir.0.057729-0

**Published:** 2013-12

**Authors:** Janet Laird, Carol McInally, Craig Carr, Sowjanya Doddiah, Gary Yates, Elina Chrysanthou, Ahmed Khattab, Andrew J. Love, Chiara Geri, Ari Sadanandom, Brian O. Smith, Kappei Kobayashi, Joel J. Milner

**Affiliations:** 1 Plant Science Research Theme, School of Life Sciences and Institute of Molecular Cellular and Systems Biology, College of Medical, Veterinary & Life Sciences, University of Glasgow, Glasgow G12 8QQ, UK; 2 Istituto di Biologia e Biotechnologia Agraria, Consiglio Nazionale Delle Richerche, Pisa, Italy; 3 School of Biological and Biomedical Sciences, Durham University, Durham DH1 3LE, UK; 4 Institute of Molecular Cellular and Systems Biology, College of Medical, Veterinary & Life Sciences, University of Glasgow, Glasgow G12 8QQ, UK; 5 Plant Molecular Biology and Virology, Faculty of Agriculture, Ehime University, Ehime 790-8566, Japan

## Abstract

Cauliflower mosaic virus (CaMV) encodes a 520 aa polypeptide, P6, which participates in several essential activities in the virus life cycle including suppressing RNA silencing and salicylic acid-responsive defence signalling. We infected *Arabidopsis* with CaMV mutants containing short in-frame deletions within the P6 ORF. A deletion in the distal end of domain D-I (the N-terminal 112 aa) of P6 did not affect virus replication but compromised symptom development and curtailed the ability to restore GFP fluorescence in a GFP-silenced transgenic *Arabidopsis* line. A deletion in the minimum transactivator domain was defective in virus replication but retained the capacity to suppress RNA silencing locally. Symptom expression in CaMV-infected plants is apparently linked to the ability to suppress RNA silencing. When transiently co-expressed with tomato bushy stunt virus P19, an elicitor of programmed cell death in *Nicotiana tabacum*, WT P6 suppressed the hypersensitive response, but three mutants, two with deletions within the distal end of domain D-I and one involving the N-terminal nuclear export signal (NES), were unable to do so. Deleting the N-terminal 20 aa also abolished the suppression of pathogen-associated molecular pattern-dependent *PR1a* expression following agroinfiltration. However, the two other deletions in domain D-I retained this activity, evidence that the mechanisms underlying these functions are not identical. The D-I domain of P6 when expressed alone failed to suppress either cell death or *PR1a* expression and is therefore necessary but not sufficient for all three defence suppression activities. Consequently, concerns about the biosafety of genetically modified crops carrying truncated ORFVI sequences appear unfounded.

## Introduction

Members of the family *Caulimoviridae* of pararetroviruses infect plants and replicate by reverse transcription of a circular dsDNA genome (Haas *et al.*, 2002). The family contains six known genera of which the most extensively studied member is cauliflower mosaic virus (CaMV), the type member of the genus *Caulimovirus*. CaMV has a genome of ~8 kb comprising six major ORFs (I–VI). Five of the six major virus proteins are translated sequentially from a single polycistronic RNA, the 35S RNA (Ryabova *et al.*, 2002, 2004). This unusual translational strategy is found in members of only two genera of viruses, *Caulimovirus* and the closely related *Soymovirus* (Ryabova *et al.*, 2002, 2006).

P6, a 62 kDa polypeptide encoded by CaMV ORFVI, was initially identified as the major component of cytoplasmic inclusion bodies, which constitute the sites of virus assembly (Haas *et al.*, 2002). P6, which is translated from its own monocistronic mRNA, plays an essential role in different aspects of virus replication. It functions in infected cells to facilitate translation of the downstream ORFs on the 35S RNA (Bonneville *et al.*, 1989; Zijlstra & Hohn, 1992) via interaction with components of the translational machinery (Bureau *et al.*, 2004; Leh *et al.*, 2000; Park *et al.*, 2001; Ryabova *et al.*, 2004), a mechanism known as translation transactivation (TAV). P6 prevents ribosome detachment at the stop codon, enabling polypeptide synthesis to reinitiate at the next start codon (Ryabova *et al.*, 2004).

At least four more roles for P6 have been identified. P6 interacts with at least two of the other CaMV proteins involved in aphid transmission, P2 and P3 (Lutz *et al.*, 2012). It forms cytoplasmic inclusion bodies of various sizes; the smaller ones associate with microtubules and the endoplasmic reticulum and move dynamically along actin filaments (Harries *et al.*, 2009). This movement is probably essential for intracellular virus trafficking and involves the interaction of P6 with the CaMV movement protein P1 (Hapiak *et al.*, 2008) and CHUP1, which mediates association between chloroplasts and the cytoskeleton. These findings suggest that P6 subverts the mechanism responsible for chloroplast movement for intracellular trafficking of CaMV (Angel *et al.*, 2013).

P6 is the major genetic determinant of virus pathogenicity (Baughman *et al.*, 1988; Kobayashi & Hohn, 2004; Schoelz *et al.*, 1986; Stratford & Covey, 1989) and expression from a transgene results in a symptom-like phenotype (Baughman *et al.*, 1988; Cecchini *et al.*, 1997; Zijlstra *et al.*, 1996). P6 exhibits virus-encoded suppressor of RNA silencing (VSR) activity (Haas *et al.*, 2008; Love *et al.*, 2007), probably through its interaction with the dsRNA-binding protein DRB4 (Haas *et al.*, 2008), a component of the Dicer4 complex.

Finally, ectopic expression of P6 in *Arabidopsis* and *Nicotiana benthamiana* profoundly affects signalling mediated by salicylic acid (SA), jasmonic acid, ethylene and auxin (Geri *et al.*, 2004; Love *et al.*, 2012; Smith, 2007). The ability of P6 to manipulate multiple components of the host defence suggests its central role as a pathogenicity determinant during virus infection, and the pleiotropic phenotypes that result from transgene-mediated expression *in planta* derive from its activity as a pathogenicity effector.

**Fig. 1.**
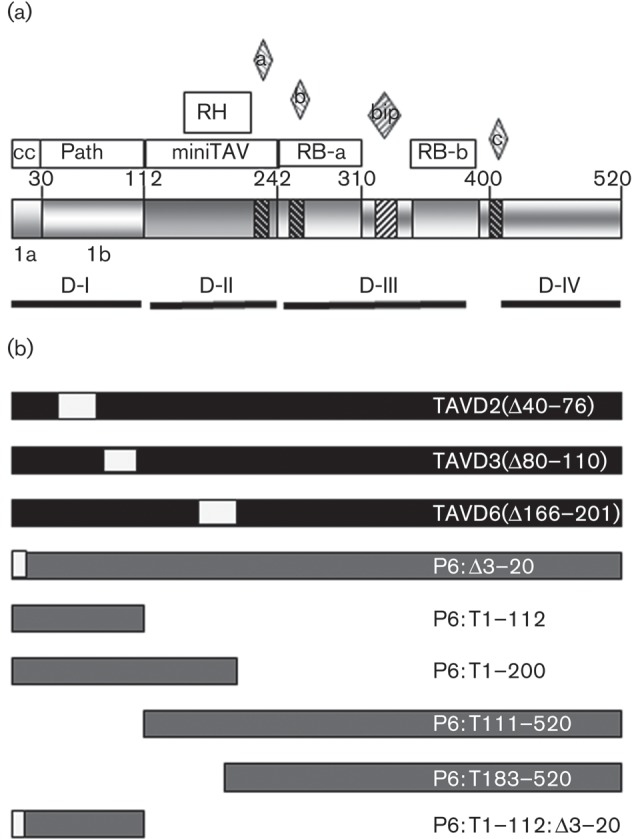
Map of the P6 domains and mutants used in this study. (a) Schematic representation of P6 domains: amino acid numbers at the boundaries of known domains are indicated. Open boxes show the coiled-coil (cc) α-helix, pathogenicity/host-range/avirulence (Path), minimum transactivator (miniTAV), RNase H (RH) and RNA binding (RB-a and RB-b). The bipartite nuclear localization signals (NLS; bip) and three non-conventional NLS (a, b and c) are indicated by diamonds above and cross-hatching. The self-association domains D-I to D-IV and subdomains 1a and 1b are indicated by solid lines. Data from Haas *et al.* (2005), Haas *et al.* (2008), Kobayashi & Hohn (2004) and Hapiak *et al.* (2008). (b) Deletions in P6 coding sequences in CaMV-TAV mutants and in the corresponding P6 expression constructs. Filled boxes indicate sequence from CaMV CM1841, shaded boxes indicate sequence derived from CaMV Cabb B-JI and open boxes indicate internal deletions.

How does a single protein achieve such a diverse range of activities? Outwith closely related members of the family *Caulimoviridae*, P6 has no obvious homologues and its three-dimensional structure is unknown. Li & Leisner (2002) defined four domains based on self-association, and sequence analysis has revealed several structural motifs and functional domains (Fig. 1a). These include RNA binding, RNase H, a short N-terminal helical domain and several predicted nuclear localization signals (NLSs) (Cerritelli *et al.*, 1998; De Tapia *et al.*, 1993; Haas *et al.*, 2008; Kobayashi & Hohn, 2003; Ryabova *et al.*, 2004). De Tapia *et al.* (1993) identified aa 111–242 (domain D-II) as containing the minimum functional domain (miniTAV domain) able to facilitate TAV. The miniTAV domain overlaps an RNase H domain (Cerritelli *et al.*, 1998) and contains the interaction motif for RL18; those for RL24, eIF4G and eIF2B are located within domain D-III (Ryabova *et al.*, 2004). Domains D-II and D-IV are involved in the interaction with CHUP1 (and presumably therefore intracellular trafficking (Angel *et al.*, 2013). P6 is a nucleocytoplasmic shuttle protein, and both nuclear localization and export functions are essential for infectivity. Mutation of a nuclear export signal (NES) at the N terminus abolishes infectivity and mutation of the predicted NLS within the C-terminal domains abolishes both VSR activity and infectivity (Haas *et al.*, 2008; Kobayashi & Hohn, 2004).

Although TAV activity and virus-trafficking functions have been mapped, the domain(s) responsible for VSR activity and SA signalling suppression remain(s) to be identified. Domain D-I plays a major role in pathogenicity and host range and acts as an avirulence domain in *Arabidopsis* and Solanaceous hosts (Agama *et al.*, 2002; Baughman *et al.*, 1988; Palanichelvam *et al.*, 2000; Palanichelvam & Schoelz, 2002; Schoelz *et al.*, 1986; Stratford & Covey, 1989). D-I has been divided into subdomain 1a, comprising the N-terminal 30 aa containing the NES (Haas *et al.*, 2008; Haas *et al.*, 2005), and subdomain 1b (aa 31–110) containing avirulence and pathogenicity functions. Mutants with deletions within 1b retain replication competence but exhibit delayed virus spread in turnip (Kobayashi & Hohn, 2004).

We carried out infection studies using CaMV mutants with deletions in P6 and found that at least one mutation within subdomain 1b abolished both VSR activity and symptom development without significantly reducing systemic virus titre. We transiently expressed WT and mutant P6 in *Nicotiana benthamiana* and *Nicotiana tabacum* and assayed the ability to suppress expression of an SA-responsive marker gene, *PR1a*, and cell death in response to a gene-for-gene elicitor. Deletions in subdomains 1a and 1b abolished VSR activity and also abolished the suppression of the cell-death response seen with WT P6. However, only the deletion in subdomain 1a eliminated suppression of *PR1a* expression. Domain D-I evidently plays an essential role in several pathogenicity effector activities. Suppression of RNA silencing and cell death may be functionally linked, but suppression of SA-responsive gene expression must involve an at least partially independent mechanism.

## Results

### Infectivity of WT and P6 deletion mutants of CaMV in *Arabidopsis*

CaMV mutants with in-frame deletions in subdomain 1b of P6 are replication competent in turnip but show delayed long-distance spread (Kobayashi & Hohn, 2004). We inoculated WT *Arabidopsis* (ecotype Col-0) with WT virus (CaMV-CW) and three mutants (Fig. 1b). CaMV-TAVD2 and CaMV-TAVD3 carry deletions in subdomain 1b, whilst CaMV-TAVD6 has a deletion in the miniTAV domain and cannot replicate in turnip.

**Fig. 2.**
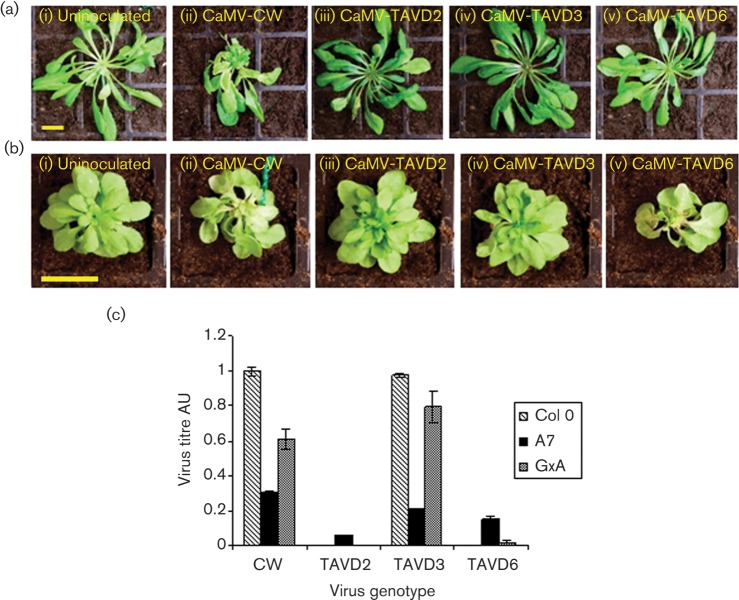
Infectivity of CaMV (WT and mutants) on WT and P6 transgenic *Arabidopsis*. (a) Symptoms on Col-0 at 28 days p.i.: (i) uninfected, (ii) CaMV-CW, (iii–v) CaMV TAV mutants as indicated. Bar, 2 cm. (b) Symptoms on P6 transgenic plants (line A7) at 28 days p.i.: (i) uninfected, (ii) CaMV-CW, (iii–v) CaMV TAV mutants as indicated. Bar, 2 cm (note difference in scale between a and b). (c) Virus titres at 28 days p.i. in Col-0, A7 and GxA plants determined by ELISA. Bars shows mean titres (±sd) of three tissue samples each comprising three pooled plants. Titres in arbitrary units (AU) are normalized to the mean of Col-0 plants infected with CaMV-CW.

Agroinoculation with CaMV-CW was remarkably efficient, with symptoms appearing at ~13 days post-infection (p.i.) and essentially 100 % of plants developing obvious stunting, leaf distortion and mosaics by 28 days p.i. (Fig. 2a). Plants inoculated with CaMV-TAVD2 and CaMV-TAVD6 did not develop any symptoms (Fig. 2a). With CaMV-TAVD3 inoculation, plants were usually asymptomatic, although by 28 days p.i. the occasional leaf exhibited subtle vein clearing. We measured virus titres at 28 days p.i. using ELISA (Fig. 2c). Col-0 plants inoculated with CaMV-CW contained high titres of virus, but CaMV-TAVD2 and CaMV-TAVD6 were not detectable by ELISA. Surprisingly, despite the lack of symptoms, titres of CaMV-TAVD3 were consistently very similar to titres of CaMV-CW. Thus, this deletion did not appear to significantly reduce virus accumulation, at least under the conditions of our experiment, but profoundly affected symptom development.

We next tested whether we could complement the mutations in ORFVI by providing P6 from the transgenic *Arabidopsis* line A7, which expresses P6 at levels similar to those in infected plants (Cecchini *et al.*, 1997) (Fig. 2b). Plants started to exhibit subtle symptoms of leaf distortion at around 14 days p.i., and by 28 days p.i., stunting and leaf distortion were visible on all A7 plants inoculated with CaMV-CW. Plants inoculated with CaMV-TAVD6 also all developed symptoms similar to CaMV-CW and at around the same time, but those inoculated with CaMV-TAVD2 and CaMV-TAVD3 did not. Titres of CaMV-CW were approximately 30 % of those in Col-0 plants, consistent with our previous reports of reduced titres in P6 transgenics (Love *et al.*, 2007, 2012). All three mutants also accumulated to significant titres (Fig. 2c). These results suggested that functional P6 provided from a transgene can act *in trans* to facilitate the replication of the mutants. ORFVI sequences provided from a transgene can under some circumstances recombine with defective CaMV genomes when infection proceeds over an extended period (Király *et al.*, 1998). Although we cannot absolutely rule out the possibility of recombination in our complementation experiments, we believe that it is unlikely because virus titres for the WT and mutants were similar at 28 days p.i. and, in the case of CaMV-TAVD6, symptoms started to appear at a similar relatively early stage in infection. The absence of symptoms in A7 plants infected with CaMV-TAVD2 and CaMV-TAVD3 suggested that, even when WT P6 is provided from a transgene, symptom development is blocked in the presence of virus-encoded P6 containing deletions in subdomain 1b. We did not observe this with CaMV-TAVD6 in which the deletion affects the miniTAV domain.

### VSR activity of CaMV deletion mutants

The transgenic *Arabidopsis* line GxA contains a 35S–GFP transgene whose expression is silenced by a second transgene, a potato virus X amplicon containing part of the GFP-coding sequence (Dalmay *et al.*, 2000; Schwach *et al.*, 2005). CaMV infection of GxA suppresses silencing of the GFP transgene, restoring strong fluorescence to infected tissue (Love *et al.*, 2007). We used this assay to compare the VSR activities of WT and mutant virus. Virus levels in GxA were similar to those in Col-0, with CaMV-CW and CaMV-TAVD3 accumulating to high titres but CaMV-TAVD2 and CaMV-TAVD6 undetectable by ELISA (Fig. 2c). As with Col-0, only CaMV-CW induced symptoms in GxA.

**Fig. 3.**
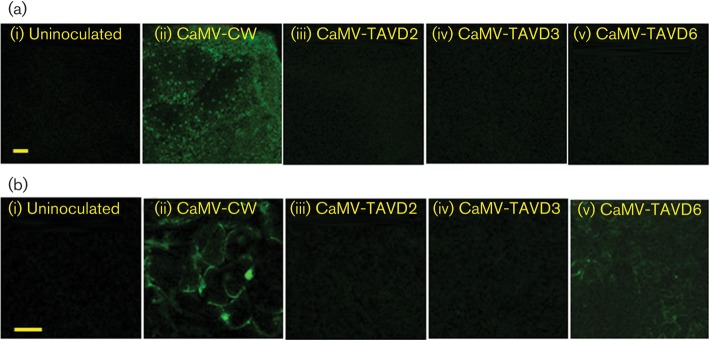
GFP fluorescence in leaves of GxA plants inoculated with CaMV WT and mutants. (a) Confocal microscope images of representative upper leaves of plants at 28 days p.i.: (i) uninoculated, (ii) CaMV-CW, (iii–v) CaMV TAV mutants. The panels show low-magnification images of GFP fluorescence. All panels were taken at the same microscope gain settings. (b) Confocal microscope images of representative inoculated leaves of plants at 28 days p.i.: (i) uninfected, (ii) CaMV-CW, (iii–v) CaMV TAV mutants. Note that the images in (b) are taken at a higher magnification than those in (a). All panels were taken with the same microscope gain settings. Bars, 100 µm.

We examined the upper leaves for GFP fluorescence using confocal microscopy (Fig. 3a). Uninoculated controls showed no detectable fluorescence, even at high gain, but tissue from CaMV-CW-infected plants consistently showed strong fluorescence. We did not observe any fluorescent cells in systemic leaves of GxA inoculated with any of the three mutants, despite the high titres of CaMV-TAVD3. To test for local silencing suppression (around the sites of inoculation), we examined inoculated leaves (Fig. 3b). With CaMV-CW, by 8–11 days p.i. we consistently observed groups of cells showing strong GFP fluorescence. Inoculated leaves recovered from the microscope slide and assayed by ELISA all contained moderate to high titres of virus (with some leaf-to-leaf variation). Titres of CaMV-TAVD3 in inoculated leaves were similar to those of CaMV-CW but we did not observe any GFP fluorescence. Leaves inoculated with CaMV-TAVD2 and CaMV-TAVD6 contained no detectable titres of virus. With CaMV-TAVD2, we did not observe any fluorescent cells, but with CaMV-TAVD6 we consistently observed fluorescence in groups of cells within every leaf we examined between 8 and 11 days p.i. (Fig. 3b), albeit at lower intensity than with CaMV-CW; by 14 days p.i. fluorescence had become undetectable. Kobayashi & Hohn (2003) showed using sensitive PCR that CaMV-TAVD6 is unable to replicate in single cells. However, agroinoculation could provide transient P6 expression through direct transcription of ORFVI (from its own 19S promoter) of the replication-incompetent CaMV-TAVD6 genome (Kobayashi & Hohn, 2003, 2004). This result demonstrates that deleting aa 166–201 does not abolish VSR activity.

### Mutations in P6 affect the ability to suppress SA-responsive cell death

**Fig. 4.**
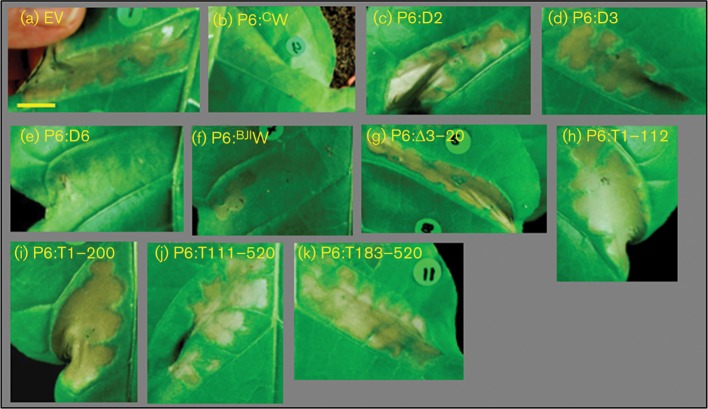
Suppression of TBSV P19-dependent cell death by co-infiltration with WT and mutant variants of P6. Photographs of leaf patches 4 days after co-agroinfiltration with a construct expressing P19 plus EV control (a) or WT or mutant P6 as indicated (b–k). All images are shown at similar magnification. Bar, 1.0 cm.

Expression of P6 from a transgene in *Arabidopsis* reduces and delays cell death following treatment with SA or inoculation with an avirulent pathogen (Love *et al.*, 2012). To identify the domain(s) responsible for this activity, we exploited the ability of tomato bushy stunt virus (TBSV) P19 to elicit a gene-for-gene hypersensitive response (HR) in *N. tabacum* in an SA-dependent manner (Angel & Schoelz, 2013; Sansregret *et al.*, 2013). *Agrobacterium*-mediated expression of P19 in *N. tabacum* gave a strong HR that was complete by 36 h but could be extended to 3–5 days by reducing the *Agrobacterium* titre to one-quarter the usual level. At the higher titre of P19, co-infiltration with a hypervirulent strain of *Agrobacterium* containing pGWB-P6^BJI^W or pGWB-P6^C^W delayed the onset of HR by approximately 24 h. At the reduced titre of P19, co-infiltration with either WT P6 construct substantially halted the progress of this HR compared with co-infiltration with empty vector (EV) (Fig. 4a, b, f). Neither WT nor mutant P6 elicited HR in the absence of P19.

We cloned the P6 coding sequences from the three CaMV mutants into Ti expression vectors and tested their ability to suppress HR (Fig. 4). In contrast to P6:^C^W (wild-type P6 from CaMV CM1841), neither of the two mutants with deletions in subdomain 1b, P6:D2 and P6:D3, was able to suppress the development of HR, but P6:D6, with a deletion in the miniTAV domain, suppressed cell death with an efficiency similar to WT (Fig. 4c–e). The N-terminal subdomain 1a contains the NES, and mutations that abolish nuclear export also abolish VSR activity (Haas *et al.*, 2008). We therefore deleted 18 of the 20 aa at the N terminus to produce what we predicted would be a functionally equivalent construct, P6:Δ3–20 (Fig. 1b). When transiently co-expressed with P19, P6:Δ3–20 was unable to suppress HR (Fig. 4g).

To test whether the D-I domain was able to suppress P19-induced HR in the absence of the C-terminal domains, we produced a further series of expression constructs (for details, see Fig. 1b). Truncated polypeptides comprising the N-terminal 112 or 200 aa (P6:T1–112, P6:T1–200) did not suppress the HR elicited by P19 (Fig. 4h, i). Neither did the corresponding C-terminally truncated variants (P6:T111–520 and P6:T183–520) (Fig. 4j, k). The ability to suppress cell death thus broadly paralleled the ability to suppress RNA silencing, with deletions in both D-I subdomains, but not in the miniTAV domain, affecting this activity. However, the D-I domain expressed alone was not sufficient to suppress cell death; therefore other regions of P6 must also be required for this activity.

### Mutations in P6 affect the ability to suppress expression of an SA-responsive marker gene

**Fig. 5.**
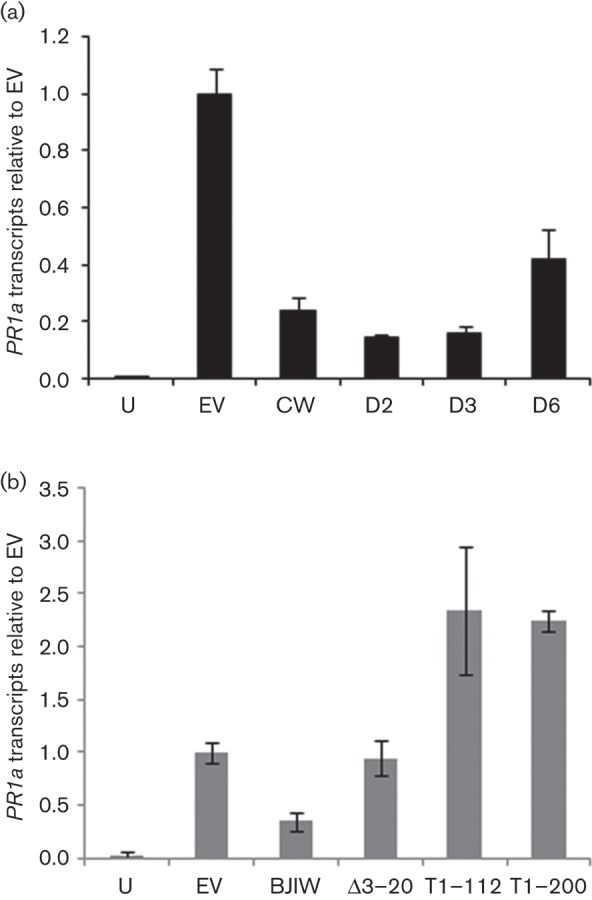
Quantification of *PR1a* expression in *N. benthamiana* leaves following transient expression of WT and mutant P6 by agroinfiltration. (a) *PR-1a* transcripts, determined by quantitative PCR, in *N. benthamiana* leaves harvested 48 h after agroinfiltration. Samples were uninfiltrated leaves (U), and leaves infiltrated with *Agrobacterium* carrying the following vectors: pGWB17 (EV), P6:^C^W (CW), P6:D2 (D2), P6:D3 (D3) and P6:D6 (D6). (b) *PR-1a* transcripts, determined as above. Samples were uninfiltrated leaves (U), and leaves infiltrated with *Agrobacterium* carrying the following vectors pGWB17 (EV), P6^BJI^W (BJIW), P6:Δ3–20 (Δ3–20), P6:T1–112 (T1–112) and P6:T1–200 (T1–200). Bars show means±sd (in arbitrary units) of three independent biological samples each comprising three pooled infiltrated leaf sections. Values were normalized to values for EV.

Agroinfiltration of *N. benthamiana* elicits pathogen-associated molecular pattern (PAMP)-responsive expression of *PR1a*, a reliable marker of SA-responsive gene expression (Volko *et al.*, 1998); this response is strongly suppressed by transient expression of P6 (Love *et al.*, 2012). P6:^C^W gave the expected reduction in *PR1a* transcripts to ~30 % that with EV (Fig. 5a). We anticipated that P6 mutants with deletions in subdomain 1b might also fail to suppress *PR1a* expression. However, P6:D2 and P6:D3 as well as P6:D6 all reduced *PR1a* transcripts to a broadly similar level to that of P6:^C^W (Fig. 5a). All three mutants evidently retained the ability to suppress SA-responsive gene expression. In contrast, infiltration with P6:Δ3–20 resulted in levels of *PR1a* expression similar to EV (Fig. 5b). Therefore, sequences required for suppression of SA-responsive gene expression are present in subdomain 1a but apparently not 1b.

We next investigated whether expressing the N-terminal domain alone was sufficient to suppress *PR1a* expression. P6:T1–112 and P6:T1–200 not only failed to reduce *PR1a* transcript levels but also produced a consistent increase of more than twofold over and above EV controls (Fig. 5b). The D-I domain is therefore not sufficient for suppression of SA-dependent gene expression but may play some role in this activity because expression on its own promoted elevated expression of *PR1a*.

### Intracellular localization of mutant P6

**Fig. 6.**
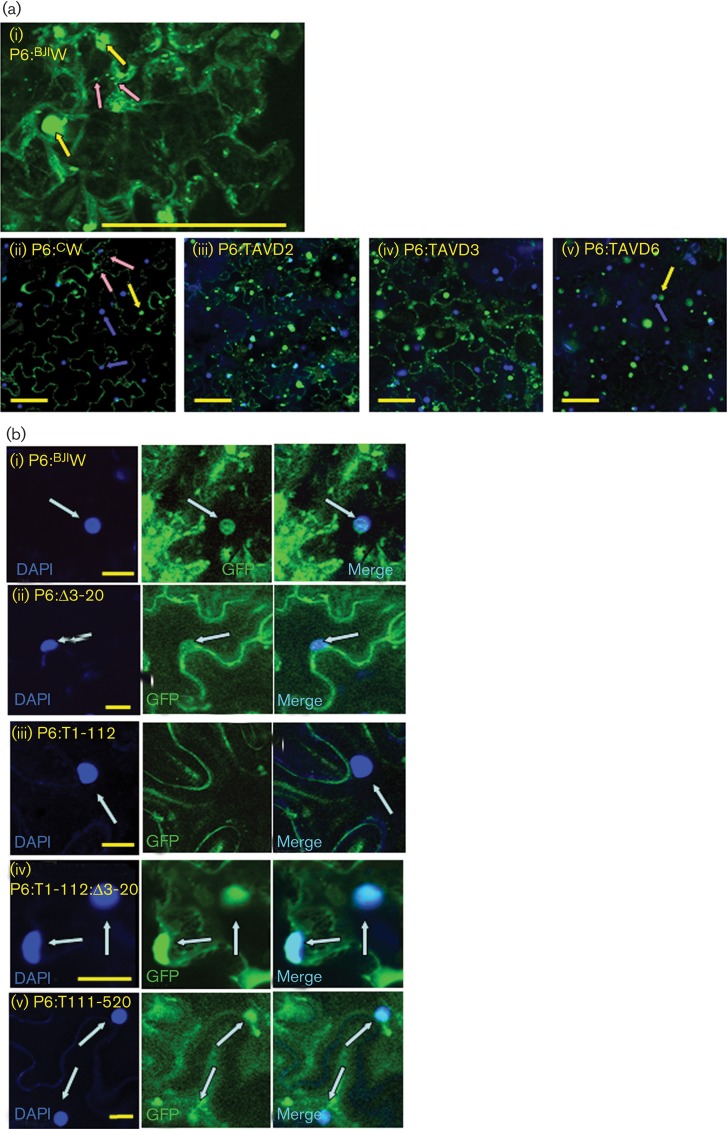
Intracellular localization of WT and mutant P6 tagged with GFP. Confocal microscope images of tissue from *N. benthamiana* leaves 3 days after agroinfiltration with WT and mutant variants of P6 fused at the C terminus to GFP. GFP fluorescence is green and DAPI fluorescence (nuclear staining) is blue. (a) Intracellular distribution of WT and TAVD mutant P6: (i) P6^BJI^W in a single epidermal cell. Representative large inclusion bodies are indicated by yellow arrows and small inclusion bodies by pink arrows. (ii) P6^C^W. Yellow and pink arrows are as in (i), whilst blue arrows indicate nuclei (DAPI staining). (iii–v) P6:D2, P6:D3 and P6:D6. Bars, 100 µm. (b) High-magnification images showing nuclear localization of WT and truncated forms of P6: (i) P6^BJI^W, (ii) P6:Δ3–20, (iii) P6:T1–112, (iv) P6:T1–112:Δ3–20 and (v) P6:T111–520. Panels from left to right: DAPI, GFP, merge. Nuclear fluorescence is indicated by arrows. Bars, 20 µm.

To test whether loss of defence suppression activity in some mutants might be attributable to mislocalization and to confirm appropriate expression of P6, we analysed its intracellular localization after transiently expressing the mutant forms of P6 as C-terminal GFP fusions in *N. benthamiana* (Fig. 6). GFP-tagged P6^BJI^W (wild-type P6 from CaMV Cabb B-JI) suppressed *PR1a* expression with an efficiency similar to a myc-tagged construct (see Methods), indicating that this activity was unaffected by the GFP tag (data not shown). Epidermal cells expressing WT P6 from the two isolates CM1841 and Cabb B-JI showed identical patterns of intracellular fluorescence, reminiscent of those reported by Harries *et al.* (2009) (Fig. 6a, i and ii). Cells contained cytoplasmic inclusion bodies that were highly variable in size. Large numbers of small inclusion bodies were present, some of which appeared to be associated with cytoplasmic strands. Large inclusion bodies were often clustered around the nucleus, but we observed only weak GFP fluorescence co-localizing with DAPI (Fig. 6b, i), consistent with the findings of Haas *et al.* (2005) who reported rapid nuclear export of P6.

Localization of P6:D2-GFP and P6:D3-GFP was indistinguishable from that of the WT (Fig. 6a, ii–iv). These deletions did not cause obvious changes in intracellular distribution. With P6:D6-GFP, we observed very few small inclusion bodies, although the large ones were still abundant (Fig. 6a, v). Domain D-II, which contains the deletion in P6:D6 (aa 166–201), has been identified as interacting with CHUP1 in connection with intracellular virus trafficking (Angel *et al.*, 2013). The region may be required for cytoskeletal association and for the formation of small inclusion bodies.

The deletion in P6:Δ3–20–GFP included three residues identified as essential for nuclear export (Haas *et al.*, 2008), so we anticipated that it would show enhanced co-localization with DAPI. Unexpectedly, the nuclear localization was similar to that of WT (Fig. 6b, ii). Haas *et al.* (2005) expressed the N-terminal 110 aa alone and compared localization with the same polypeptide with the N-terminal α-helix deleted. We produced equivalent constructs (although with a C-terminal GFP tag), P6:T1–112–GFP and P6:T1–112:Δ3–20–GFP. Whereas P6:T1–112–GFP showed no nuclear localization whatsoever, P6:T1–112:Δ3–20–GFP co-localized strongly with DAPI (Fig. 6b, iii, iv). P6:T111–520–GFP, which lacks the entire D-I domain (including the NES), also showed enhanced nuclear localization (Fig. 6, v). Our results are therefore consistent with deletion of the N-terminal 20 aa affecting nuclear export. Haas *et al.* (2005) fused GFP to the N terminus; our use of a C-terminal tag might account for the differences for full-length P6.

### Sequence conservation of domain D-I across members of the *Caulimoviridae*

**Fig. 7.**
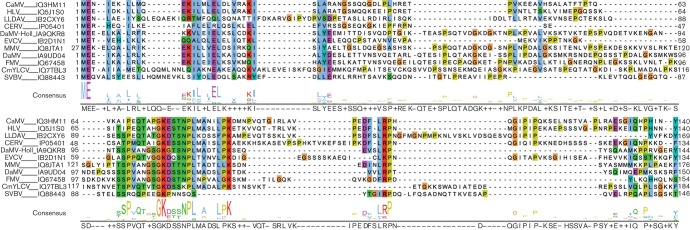
Alignment of sequences of the N-terminal amino acids of P6 from caulimoviruses. The sequences from CaMV, horseradish latent virus (HLV), lamium leaf distortion associated virus (LLDAV), carnation etched ring virus (CERV), dahlia mosaic virus-Holland (DaMV-Holl), eupatorium vein clearing virus (EVCV), mirabilis mosaic virus (MMV), dahlia mosaic virus (DaMV), figwort mosaic virus (FMV), cestrum yellow leaf curling virus (CmYLCV) and strawberry vein banding virus (SVBV) that precede the RNaseH domain (aa 140 in CaMV) are aligned, with the consensus sequence shown below in logo form. Residues are coloured according to the clustal_x colouring scheme and the Uniprot accession numbers indicated.

We used the programs Jalview (Waterhouse *et al.*, 2009) and JPred3 (Cole *et al.*, 2008) to align the sequences of P6 from CaMV with ten other members of the genus *Caulimovirus* and four members of the genus *Soymovirus*es. P6 from all ten caulimoviruses showed significant homology with CaMV over almost the entire sequence, but none of the soymoviruses showed significant similarity to the caulimovirus D-I domain. (Figs 7 and S1, available in JGV Online). Within domain D-I, homology varied between different members of the genus *Caulimovirus* (Fig. 7), but there was notable sequence conservation between aa 67 and 88, in particular the GK(D/E)X(S/T)NPLXXXXLXK motif (aa 74–88) conserved in 10 of 11 sequences. Interestingly, this motif extends across the junction between the TAVD2 and TAVD3 deletions.

The N-terminal sequences of the soymoviruses are shorter than those of the caulimoviruses and are rather diverse (Fig. S1). Possibly, members of the genus *Soymovirus* lack a functional D-I domain.

## Discussion

We have shown that the N-terminal domain of P6 contains sequences essential for its activities as a suppressor of RNA silencing and of SA-dependent defence responses. These appear to be distinct from its TAV and virus-trafficking functions. Deleting the distal end of subdomain 1b (aa 80–110) abolished VSR activity within the context of an infectious virus clone. The same deletion also abolished the ability to suppress one aspect of SA-dependent signalling, cell death triggered by the elicitor TBSV P19, but not another, PAMP-driven *PR1a* expression. The mechanisms underlying these three activities may therefore overlap but are clearly not identical. However, the N-terminal subdomain 1a, which includes the NES, is essential for all three.

CaMV-TAVD6, with a deletion within the miniTAV domain, consistently produced a transient silencing suppression in inoculated leaves, evidence that this mutant retains VSR activity. As CaMV-TAVD6 is completely unable to replicate in protoplasts (Kobayashi & Hohn, 2003), we assume that P6 mRNA is transcribed directly from CaMV-TAVD6 genomes introduced by agroinoculation. Although we were unable to detect CaMV-TAVD2 accumulation in inoculated leaves using ELISA, we might have expected similar limited P6 expression by direct transcription of the T-DNA following agroinoculation. If so, the failure of CaMV-TAVD2 to stimulate similar transient GFP expression in inoculated leaves suggests that it too may be deficient in VSR activity.

Suppression of RNA silencing by VSR is a major contributor to symptom induction (Burgyán & Havelda, 2011). Despite titres similar to WT virus, CaMV-TAVD3 was essentially asymptomatic on Col-0 plants, suggesting that the symptoms of CaMV infection (at least in *Arabidopsis*) are probably linked to the VSR activity of P6. Complementation by transgene-derived P6 allowed all three mutants to replicate, but, whereas CaMV-CW and CaMV-TAVD6 caused obvious stunting and leaf distortion in A7, CaMV-TAVD3 and CaMV-TAVD2 were both asymptomatic. As A7 produces high levels of WT P6 (Cecchini *et al.*, 1997), the absence of symptoms in CaMV-TAVD2- and CaMV-TAVD3-infected plants must be a dominant-negative effect, presumably linked to the loss of VSR activity, further evidence that CaMV-TAVD2 is also defective in this respect.

A role for domain D-I in defence suppression is consistent with its identification as the major genetic determinant of virus pathogenicity, host range and avirulence (Kobayashi & Hohn, 2003; Palanichelvam & Schoelz, 2002; Schoelz & Shepherd, 1988; Stratford & Covey, 1989). Both subdomains are involved. Subdomain 1a clearly plays an essential role in pathogenicity as deleting it eliminated both the suppression of cell death and PAMP-responsive gene expression. Correct localization of P6 may be required for these activities. Deletions within subdomain 1b abolished the ability to suppress cell death in our assay but not the ability to suppress PAMP-triggered expression of *PR1a*. The apparent discrepancy between the effects of deletions in subdomain 1b on these two different SA-dependent responses may be explained by the recent report that extreme resistance to TBSV in *N. tabacum* is elicited by a complex of P19 plus small interfering RNAs (siRNAs) (Sansregret *et al.*, 2013). Interaction with DRB4, a component of the Dicer4 complex (Haas *et al.*, 2008), provides a probable mechanism for the VSR activity of P6. As Dicer4 is involved in generating siRNAs, both defence suppression activities of P6 (on RNA silencing and SA signalling) could potentially play a role in inhibiting the HR elicited by P19.

The truncated proteins P6 : 1–112 and P6 : 1–200 elicited elevated levels of *PR1a* transcripts compared with EV controls. The C-terminal region of P6, which is absent from these constructs, contains four predicted NLSs (Fig. 1a). Nuclear localization is required for VSR activity (Haas *et al.*, 2008), and our results are consistent with it also being essential for cell death and suppression of SA-dependent gene expression. We did not observe obvious differences in nuclear localization between WT and truncated proteins (Fig. 6). However, because the N-terminal NES promotes very efficient re-export of P6 from the nucleus, even WT P6, which is actively imported into the nucleus (Haas *et al.*, 2005), gave only weak GFP fluorescence within nuclei.

The effects of mutations in subdomain 1b on VSR activity and the suppression of the HR elicited by P19 imply that it must play a key role in these functions. The motif GK(D/E)X(S/T)NPLXXXXLXK, which spans the ends of the TAVD2 and TAVD3 deletions, is very highly conserved across 10/11 members of the genus *Caulimovirus*. Such a degree of sequence homology provides additional support for the importance of this region of P6 to members of this genus.

The pleiotropic phenotype(s) of P6-transgenic *Arabidopsis* (Geri *et al.*, 2004; Love *et al.*, 2012; Smith, 2007) would be most elegantly accounted for by a common underlying mechanism, perhaps all involving RNA silencing, rather than by diverse direct interactions with multiple signalling intermediates. However, because deletion mutants of subdomain 1b suppressed PAMP-responsive *PR1a* expression with a similar efficiency to WT P6, VSR activity must not be essential for this activity.

Transgene-mediated expression of VSRs elicits pleiotropic effects on jasmonic acid and other phytohormone responses (Endres *et al.*, 2010; Lewsey *et al.*, 2010; Lozano-Durán *et al.*, 2011; Yang *et al.*, 2008), and CaMV infection is accompanied by profound changes in microRNA (miRNA) and *trans*-acting siRNA (tasiRNA) populations (Blevins *et al.*, 2006; Moissiard & Voinnet, 2006; Shivaprasad *et al.*, 2008). The 5′ leader sequence of the CaMV 35S RNA is a target for all four *Arabidopsis* Dicer complexes, producing siRNAs that appear to target host transcripts (Blevins *et al.*, 2006; Moissiard & Voinnet, 2006; Shivaprasad *et al.*, 2008), evidence of a complex interaction mediated at least partially by RNA silencing. miRNAs and tasiRNAs regulate signalling pathways involving auxin (Rubio-Somoza & Weigel, 2011; Rubio-Somoza *et al.*, 2009), ethylene (Pei *et al.*, 2013) and jasmonic acid (Lewsey *et al.*, 2010; Schommer *et al.*, 2008; Zhang *et al.*, 2012), and evidence is emerging that they also regulate immune responses and cell death in *Arabidopsis* (Alonso-Peral *et al.*, 2010; Li *et al.*, 2012).

We previously identified NPR1, a central regulator of defence, as a target for P6. NPR1 acts through complex mechanisms entailing activation by SA, nuclear localization, modification (phosphorylation and *S*-nitrosylation) and targeted proteolysis (Mukhtar *et al.*, 2009). Recent reports identify three SA receptors, NPR1 itself (Wu *et al.*, 2012) plus two E3 ligases, NPR3 and NPR4. These suppress or activate both programmed cell death and *PR* gene expression by regulating NPR1 levels in response to changes in intracellular SA (Fu *et al.*, 2012). Expression of P6 alters intracellular localization and enhances accumulation of NPR1 (Love *et al.*, 2012). The possibility that this might be achieved through miRNAs or tasiRNAs is intriguing. All four *Arabidopsis* Dicers are believed to participate in the biogenesis of siRNAs from the CaMV leader (Blevins *et al.*, 2006; Moissiard & Voinnet, 2006), but it is DCL1 that is primarily responsible for the generation of miRNAs from host-encoded precursors (Vazquez *et al.*, 2010). It would be interesting to investigate whether P6 and HYL1 (the DRB4 homologue in Dicer1) also interact. Although the details of the mechanisms remain unknown, our results suggest that RNA silencing regulates at least one response involving SA signalling (cell death), and that CaMV targets multiple defence responses via the VSR activity of P6.

Fifty-four transgenic events commercialized in the USA contain up to 528 bp of the coding region of ORFVI (Podevin & du Jardin, 2012). The potential expression of a C-terminal P6 polypeptide with defence-suppressing properties has been identified as a possible hazard in genetically modified crops (Latham & Wilson, 2013). The essential role for the N-terminal region of P6 in these activities demonstrates that these concerns are unfounded.

## Methods

### 

#### Virus infection.

*Arabidopsis* plants were grown under short days as described previously (Cecchini *et al.*, 1998). Details of the P6-transgenic line A7 have been published (Cecchini *et al.*, 1997). For assaying VSR activity, we infected transgenic line GxA in which expression of GFP is silenced by a potato virus X amplicon (Dalmay *et al.*, 2000; Love *et al.*, 2007; Schwach *et al.*, 2005).

Virus infection was achieved using agroinfectible constructs derived from WT CaMV isolate CM1841 (pFastWt) and its ORFVI mutants, pFastTavD2, pFastTavD3 and pFastTavD6 (Kobayashi & Hohn, 2003, 2004; Tsuge *et al.*, 1994), which were designated in this study as CaMV-CW, CaMV-TAVD2, CaMV-TAVD3 and CaMV-TAVD6, respectively. Full details of the construction of the agroinfectible clones are given in Fig. S2.

For virus infection, the hypervirulent *Agrobacterium* strain AGL1+virG (Vain *et al.*, 2004) containing the appropriate construct was grown overnight at 28° in Luria–Bertani medium containing kanamycin (50 µg ml^−1^), rifampicin (50 µg ml^−1^) and gentamicin (50 µg ml^−1^). Bacteria were resuspended at OD_600_ = 0.2 in 10 mM MgCl_2_ and incubated for 2 h with 200 µM acetosyringone at room temperature. Celite was added, and plants at the eight-leaf stage were inoculated by pipetting 2 µl bacterial suspension onto one lower leaf and rubbing with a sterile inoculating loop.

Virus titres were measured using DAS-ELISA kits (Bioreba, Lynchwood Diagnostics, UK). The entire above-ground parts of three infected plants were combined, ground in 10 vols of Extraction Buffer (Bioreba), clarified in a bench-top centrifuge and the supernatant assayed according to the manufacturer’s instructions. Samples with high virus titres were diluted a further 10-fold before assay.

#### Transient expression of WT and mutant variants of P6.

Transient expression was carried out by agroinfiltration in *N. benthamiana* as described previously (Bazzini *et al.*, 2007; Love *et al.*, 2012). Vectors for expressing WT or mutant P6 were constructed using the Gateway cloning system (Invitrogen). Sequences were amplified by PCR using the primer combinations listed in Table S1, inserted into pENTR-DTopo and transferred to Gateway binary vectors pGWB17 (giving a C-terminal 4×myc tag) or pGWB5 (giving a C-terminal GFP fusion). Details of the deletions and truncations are shown in Fig. 1(b). Constructs were derived from CaMV isolate CM1841 (GenBank accession no. V001440) or the closely related Cabb B-JI (GenBank accession no. DQ211685). Details of pGWB-P6^BJI^W have been described in Love *et al.* (2012; referred to as pGWB-P6myc). p35S-P19 for expression of TBSV P19 (Voinnet *et al.*, 2003) was a kind gift from Professor David Baulcombe (Cambridge, UK).

For the cell death suppression assay, TBSV P19 and P6 were co-expressed in leaves of *N. tabacum* (cv. Petite Havana SR1). Overnight cultures of p35S-P19 in *Agrobacterium* GV3101, were resuspended at OD_600_ = 0.1. *Agrobacterium* AGL1+virG containing the appropriate P6 expression construct (or as control pGWB17) were resuspended at OD_600_ = 0.4 and mixed with an equal volume of the P19 culture for infiltration. The development of necrosis was assessed visually over 3–5 days. To allow for potential leaf-to-leaf differences in the development of HR, we always included one WT P6 as a positive control and one EV as a negative control on each leaf.

#### Quantification of transcripts by quantitative PCR (qPCR).

*NbPR1a t*ranscripts were quantified by real-time reverse transcription qPCR using a Stratagene MX4000 or MX3000 thermocycler as described previously (Love *et al.*, 2005, 2012). The reference gene was *NbEF1*α. Each biological sample comprised RNA extracted from ~50 mg tissue taken from the infiltrated area of a single *N. benthamiana* leaf. The primers are given in Table S1(B).

#### Fluorescence microscopy.

GFP fluorescence in leaves of GxA and localization of P6–GFP in *N. benthamiana* leaves were followed using a Zeiss LSM510 confocal microscope essentially as described previously (Love *et al.*, 2007, 2012). Nuclei were stained with DAPI (Molecular Probes, Life Technologies).

#### Protein sequence alignments.

The Jpred3 server was searched with the CaMV sequence (NCBI Protein no. Q3HM11) to obtain an alignment of diverse P6 sequences with redundancy removed. The selected sequences were retrieved intact from the Uniprot database and the alignment rebuilt with muscle (Edgar, 2004) and curated manually in Jalview.
